# Impacts of heatwaves on electricity reliability: Evidence from power outage data in China

**DOI:** 10.1016/j.isci.2025.111855

**Published:** 2025-01-21

**Authors:** Jing Liang, Yueming (Lucy) Qiu, Bo Wang, Xingchi Shen, Shangwei Liu

**Affiliations:** 1 School of Management, Harbin Institute of Technology, Harbin, China; 2 School of Public Policy, University of Maryland College Park, College Park, MD, USA; 3 School of Management and Economics, Beijing Institute of Technology, Beijing, China; 4 School of Environment, Yale University, New Haven, CT, USA; 5 School of Public and International Affairs, Princeton University, Princeton, NJ, USA

**Keywords:** Global change, Energy systems, Energy Modelling

## Abstract

Heatwaves, driven by climate change, have increasingly challenged energy systems with increased demand and reduced supply, leading to power outages. This study empirically examines the impact of heatwaves on power outages, employing fixed-effects models and using high-frequency outage data from China (2019–2021). The results indicate that heatwaves increase the frequency of outages by 3.9%–4.0% and extend their duration by 7.9%–8.3%. Additionally, each degree of temperature rise increases outages by 0.1%, and an additional heatwave day raises outages by 0.5%. We also observed heterogeneity in outage impacts across different socio-demographic groups. Furthermore, projections under RCP2.6, RCP4.5, and RCP8.5 show that outages will increase by 5.2%–12.5% in 2030 and 7.4%–20.3% in 2050. These findings underscore the urgency of grid upgrades and provide insights for resource allocation to adaptation to climate change.

## Highlights

• Outages occur during heatwaves due to surging demand and reduced supply
• Heatwaves increase both the frequency and duration of outages in China
• There is heterogeneity in outage impacts across socio-demographic groups
• We projected outages under three future scenarios: RCP2.6, RCP4.5, and RCP8.5

## Graphical abstract

**Figure:**
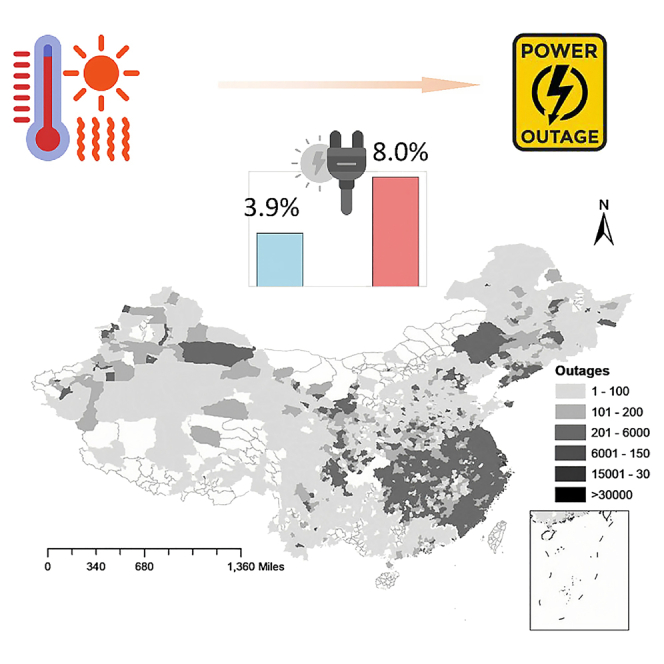

## Introduction

Globally, climate change has significantly increased the frequency of extreme weather events, and heatwaves occur more often even in temperate areas.^1^ In 2022, deadly heatwaves struck India, while European countries such as the UK and Italy also experienced growing intensity and duration of heatwaves.^2^^,^^3^ In South Asia, heatwave events were reported to be 30 times more likely due to climate change.^4^ Concurrently, China endured record-breaking and prolonged heatwaves in 2022, unprecedented since the last major event a decade earlier. Studies have shown that extreme heat events adversely affect human health and damage economic and agricultural production.^5^^,^^6^^,^^7^^,^^8^^,^^9^ Furthermore, heatwaves also impact energy systems by affecting both electricity supply and demand. High temperatures escalate cooling demand with the penetration of air conditioning. Simultaneously, heatwaves compromise supply. Hydroelectric power is constrained, efficiency is jeopardized due to insufficient cooling water, and the transmission capacity is impaired.^10^^,^^11^^,^^12^^,^^13^ Despite the insights on how demand for cooling surges, and on how energy supply is constrained,^14^^,^^15^^,^^16^ there remains a gap in comprehensive quantification of these two impacts. The empirical evidence linking heatwaves to outages is important given that weather-induced power outages have extensive consequences,^17^^,^^18^^,^^19^^,^^20^^,^^21^ particularly for the vulnerable communities susceptible to heat-related illnesses.^19^^,^^20^ Our study fills such a gap by providing an empirical assessment of the electricity supply reliability in China, applying high-frequency power outage data. Such studies become particularly critical during frequent or prolonged extreme weather events.

Heatwaves compromise the operation of the energy infrastructure and grid systems. Grid systems may be designed based on the historical magnitude of extreme events while grid infrastructure components constructed decades ago have degraded over time.^2^^,^^22^ The increasing integration of renewable energy into the grid could also increase its vulnerability to disturbances.^23^ Previous studies on the resilience of power system design through modeling and simulations^24^^,^^25^^,^^26^^,^^27^^,^^28^; however, these studies often are missing an empirical perspective.^21^^,^^22^^,^^23^^,^^24^^,^^25^ The empirical evidence linking heatwaves and outages is therefore essential for developing more resilient energy systems.

**Figure 1:**
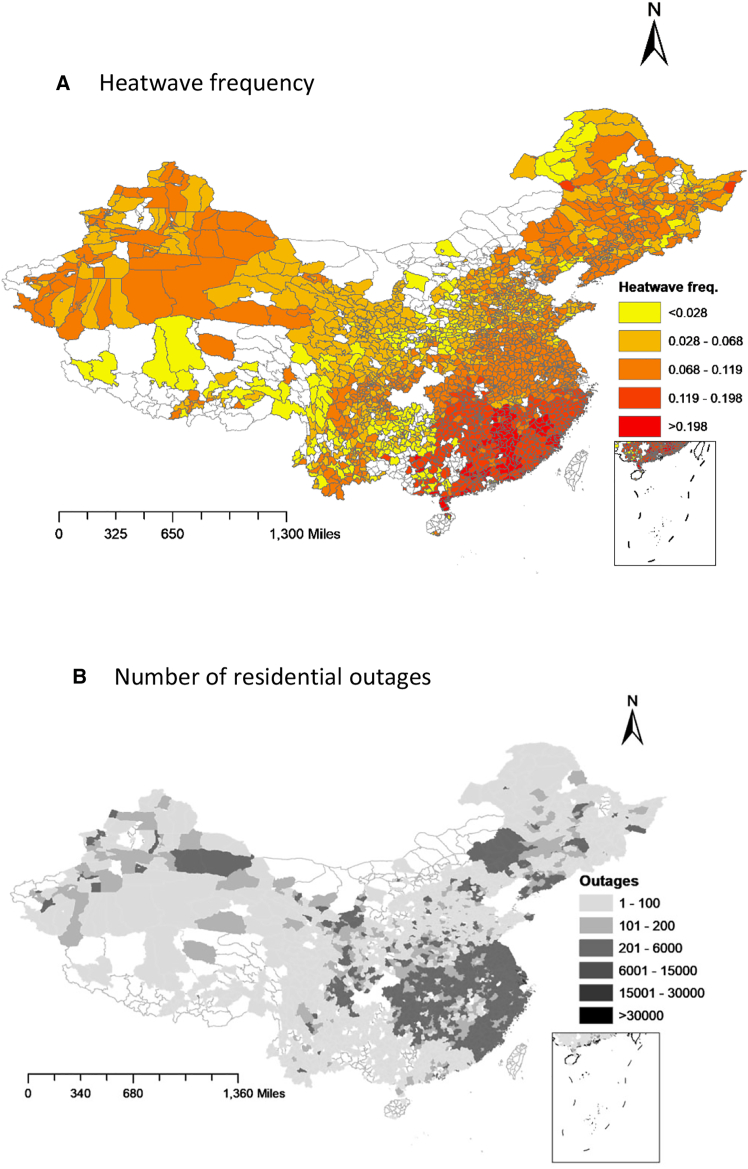
Maps of heatwaves and outage events in China during 2019–2021 The data are plotted at the county level. Heatwave frequency in (A) indicates the proportion of heatwave days to total days. The number of outages (in incidents) in (B) is the sum of outages for each county during the study period. Blank areas indicate missing data.

Heatwaves are generally characterized as high-temperature events spanning several consecutive days, although the appropriate heatwave threshold varies across different regions.^1^^,^^29^^,^^30^^,^^31^^,^^32^ We define heatwaves as periods of at least three consecutive days during which the daily mean temperature falls within the highest 10% of the temperature distribution.^30^^,^^31^^,^^33^ We incorporated an alternative definition in the robustness checks. This study aims to quantify the increase in the probability of electricity outages in response to extreme heatwave events in China (Figures 1 and S1), using data from about 2,800 county-level administrative divisions from 2019 to 2021.

We first matched the meteorological records to county-level outage data, using an inverse distance weighting method.^34^^,^^35^^,^^36^ Then, we estimated the causal effect of heatwaves on outages using panel regressions. The regression models include a set of fixed effects and covariates to control for confounding factors and address omitted variable issues. Additionally, we investigated how heatwave intensity and duration influence outages, explored variations in outage responses across different socio-demographic groups, and examined the effect of proximity to power plants on outage frequency. Furthermore, we also assessed how heatwave-induced outages might escalate under three representative concentration pathway (RCP) scenarios: RCP2.6, RCP4.5, and RCP8.5. Moreover, our robustness checks included an alternative definition of heatwaves, an examination of only unplanned outages, and an analysis excluding other environmental hazards. The findings of this study are crucial for energy planning authorities to prepare for future electricity shortages and underscore the need for timely investments in grid infrastructure upgrades to mitigate adverse effects. Furthermore, the identified heterogeneity in outage impacts provides valuable insights into which vulnerable communities should be prioritized for interventions and resource allocation.

## Results

### Heatwave-induced outages

We examine whether heatwaves lead to more frequent and longer power outages. Using panel fixed effects regressions, we examine the impact of heatwave events on power outages in terms of both frequency and duration. The econometric models allow us to identify the causal changes in power outages in response to heatwaves after controlling for confounding factors (Methods S1). Specifically, we explore the within-county variation by comparing outages during heatwaves to those not during heatwaves. A series of fixed effects and covariates is included in the model to eliminate the confounding effects from county-specific time-invariant factors and certain time-varying variables. Details of the model specification are shown in the STAR Methods section.

Our results (Table 1) indicate that heatwaves increase the frequency of electricity outages by 3.9%–4.0%. Heatwaves also extend the length of power outages by 7.9%–8.3%. Column (1) includes county- and year-fixed effects (a set of county and year indicator variables), while columns (2) and (3) include city-by-year fixed effects and county-by-year fixed effects, respectively. Column (4) clusters the standard errors at the prefectural city level, while the other columns cluster at county-level administrative divisions (there are altogether about 300 prefecture-level cities and about 3,000 county-level administrative divisions in China). The results across different columns are very similar to each other. Summary statistics are provided in Table S1, and the full regression results are in Table S2.

**Table 1:** Impacts of heatwaves on power outages

	(1)		(2)		(3)		(4)	
	Outage occurrence	Outage length	Outage occurrence	Outage length	Outage occurrence	Outage length	Outage occurrence	Outage length
Heatwave	0.040∗∗∗ (0.002)	0.083∗∗∗ (0.006)	0.039∗∗∗ (0.002)	0.080∗∗∗ (0.006)	0.039∗∗∗ (0.002)	0.079∗∗∗ (0.006)	0.040∗∗∗ (0.005)	0.083∗∗∗ (0.011)
County fixed effects	Yes	Yes	No	No	No	No	Yes	Yes
Month fixed effects	Yes	Yes	Yes	Yes	Yes	Yes	Yes	Yes
City-by-year fixed effects	No	No	Yes	Yes	No	No	No	No
County-by-year fixed effects	No	No	No	No	Yes	Yes	No	No
Weather covariates	Yes	Yes	Yes	Yes	Yes	Yes	Yes	Yes
Climate extreme covariates	Yes	Yes	Yes	Yes	Yes	Yes	Yes	Yes
Clustered at the city	No	No	No	No	No	No	Yes	Yes
Number of counties	2,722	2,722	2,722	2,722	2,722	2,722	2,722	2,722
N	803,746	803,746	803,746	803,746	803,746	803,746	803,746	803,746
R^2^	0.067	0.062	0.076	0.072	0.089	0.084	0.067	0.062

The dependent variable is the logarithm of outages. County-by-year fixed effects control for the county-specific unobserved factors across years. Month fixed effects control for temporal variation using month indicators. Standard errors in parentheses are clustered at the county level unless otherwise specified. Clustering standard errors at the city level provides cluster-robust standard errors that reflect the data clustering within a city. ∗*p* < 0.10, ∗∗*p* < 0.05, ∗∗∗*p* < 0.01.

The outages are probably caused by the fault of weakly meshed distribution network, especially the failures in electric components such as underground cable, substations, bus-bars, and transformers.^2^^,^^37^ Research has observed the correlation between heatwaves and medium voltage underground cable faults.^37^^,^^38^^,^^39^ Abnormal faults in underground cable joints are reported to be concentrated on hot days,^39^^,^^40^^,^^41^ as high temperatures expose components to excessive heat, making heat dissipation difficult^2^^,^^3^ (Section S1).

We also provide a back-of-the-envelope analysis of the economic losses and compare them to the costs of grid upgrades. This estimation is crucial given the significant investments and projected expansion of the existing grid. For instance, China has been investing over 450 billion CNY (Chinese Yuan) annually since 2020. The estimations are based on the following assumptions: (1) the average economic losses for consumers are 70.9 yuan/kWh, as detailed in Table S3, with alternative outage costs in Table S4; (2) the annual per capita electricity consumption is 5,300 kWh, according to the China Energy Statistical Yearbook (2020–2022)^42^; and (3) the average population per county is estimated at 500 thousand.^43^ The total economic losses associated with heatwave-induced outages are estimated at 23.3 billion yuan (or 3.4 billion USD) annually per county. Such losses are equivalent to 0.78 billion yuan per heatwave event given the average incidence of about 30 heatwave events annually per county.

In contrast, the annual costs for upgrading the grid infrastructure are estimated at 4.05 billion yuan per county. This estimate assumes that the marginal cost for improving supply reliability is approximately 16.2 yuan per consumer per minute of interruption, as detailed in Table S5.^44^ The comparison of the losses with the costs reveals that the economic losses are greater than the costs of grid upgrades, underscoring the cost-effectiveness of timely investments in enhancing grid infrastructure and bolstering system reliability.

### Effects of heatwave intensity and duration

Our analysis (Table 2, column 1) shows that for each degree Celsius increase in temperature during a heatwave, the frequency and duration of electricity outages both increase by about 0.1%. Furthermore, the duration of heatwaves significantly exacerbates these outages; each additional day of a heatwave increases the occurrence of outages by 0.5% and their duration by 1.1% (column 2). The results suggest that longer heatwaves increase the disruptions caused by heatwaves and cause more frequent failures of electricity supply.

**Table 2:** Impacts of heatwaves intensity and duration on power outages

	(1)		(2)	
	Outage occurrence	Outage length	Outage occurrence	Outage length
Heatwave intensity	0.001∗∗∗ (0.00007)	0.001∗∗∗ (0.0002)		
Heatwave duration			0.005∗∗∗ (0.0005)	0.011∗∗∗ (0.001)
County-year fixed effects	Yes	Yes	Yes	Yes
Month fixed effects	Yes	Yes	Yes	Yes
Weather covariates	Yes	Yes	Yes	Yes
Climate extreme covariates	Yes	Yes	Yes	Yes
Number of counties	2,722	2,722	2,722	2,722
N	803,746	803,746	803,746	803,746
R^2^	0.089	0.036	0.089	0.084

The dependent variable is the logarithm of outages. Standard errors are in the parentheses and are clustered at the county level. ∗*p* < 0.10, ∗∗*p* < 0.05, ∗∗∗*p* < 0.01.

### Heterogeneity among socio-demographics

As the conditions of electricity supply and demand exhibit notable variations across different socio-demographic groups, we explore how the impacts of heatwave-induced outages differ among these groups. We explore the disparities in heatwave-induced power outages across three socioeconomic indicators: per capita income, population density, and primary industry production levels. We categorize the data into three strata for each characteristic and conduct separate regression analyses for each level.

The impacts of socio-demographics on outages are mixed. While regions with lower income and more agricultural production tend to be less developed, and consumers often more socioeconomically disadvantaged and face more power outages due to inferior infrastructure, their electricity consumption, on the other hand, is also limited by their financial constraints, which could dampen the increase in electricity demand during heatwaves. If the latter effect predominates, we would expect smaller impacts on the lower-income group.

**Figure 2:**
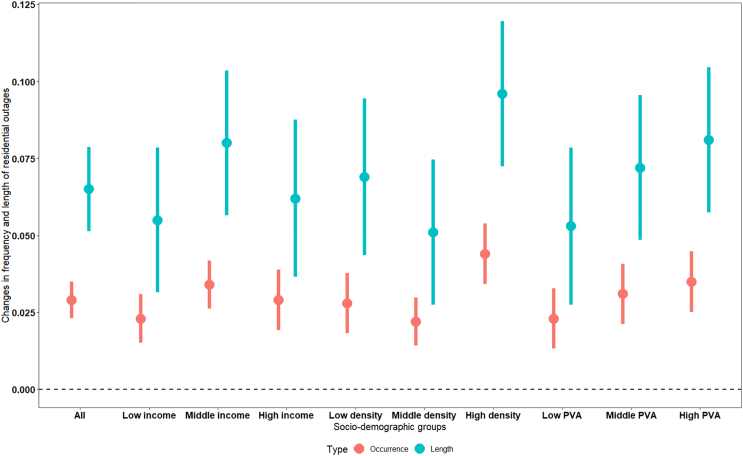
Heterogeneous impacts of heatwaves on power outages by different socio-demographics The solid dots denote the coefficients that estimate the mean impacts, and the lines refer to the 95% confidence intervals of the coefficients. PVA (primary industry value added) measures the changes in value added by the primary industry sector (agricultural sector) to the total GDP.

The results are depicted in Figure 2. The middle-income households are more susceptible to electricity outages during heatwaves compared to high-income counterparts, which likely benefit from greater investments in electrical infrastructure.^45^ Surprisingly, low-income households are also less responsive to heatwaves compared to middle-income ones, possibly due to limited access to air conditioning and less elasticity in demand driven by income constraints.^15^^,^^46^ Additionally, our study finds that the most populous areas are more prone to outages than less densely populated ones, a phenomenon possibly linked to increased demand pressures in more populated regions. Additionally, regions with higher levels of primary industrial production are more susceptible to heatwave-induced outages. This suggests that these areas, typically rural and with inadequate grid and power infrastructure, may be particularly vulnerable to disruptions.^47^^,^^48^

The socio-demographic data are sourced from the China County Statistical Yearbook.^49^ This subsample of rural counties enables a targeted exploration of the specific responses within more rural communities. We further incorporated aggregated social-demographic data for all the prefecture-level cities and conducted comparable analyses for both urban and rural settings (Figure S2). The findings align with Figure 2, but the larger coefficients for the three income groups indicate income’s greater impact on the general population compared to the rural subgroup.

### Distance to power plants

Given that heatwaves diminish electricity supply by reducing transmission capacity, this section explores the effect of transmission distance from power plants on heatwave-induced power outages. We focus specifically on coal power plants, as they are the predominant source in China’s power mix, accounting for approximately 65% of electricity generation and much of the distribution infrastructure is centered around these facilities.^50^^,^^51^ We first obtained data on coal power plants from the Global Coal Plant Tracker and measured the distance between cities and power plants. Then, we incorporated the distance to coal power plants into our regression analysis.

Our results (Table 3) demonstrate that power outages during heatwaves are positively correlated with the distance from power plants. This trend can be attributed in part to the necessity for longer transmission lines as the distance from power plants grows. Specifically, for every 100 km farther away from power plants, the occurrence of outages increases by 3.9%, while their length increases by 8.9%. These results highlight the need for reconstruction and upgrading of grid infrastructure in distant areas to enhance the resilience of energy systems against the impacts of climate change.

**Table 3:** Impact of supply distance on heatwave-induced outages

	(1)		(2)	
	Outage occurrence	Outage length	Outage occurrence	Outage length
Heatwave	0.027∗∗∗ (0.003)	0.053∗∗∗ (0.007)	0.026∗∗∗ (0.003)	0.049∗∗∗ (0.007)
Heatwave∗ distance to CPP	0.039∗∗∗ (0.006)	0.089∗∗∗ (0.012)	0.039∗∗∗ (0.005)	0.089∗∗∗ (0.012)
County fixed effects	Yes	Yes	No	No
Year fixed effects	Yes	Yes	No	No
Month fixed effects	Yes	Yes	Yes	Yes
County-by-year fixed effects	No	No	Yes	Yes
Number of counties	2,722	2,722	2,722	2,722
Weather covariates	Yes	Yes	Yes	Yes
Climate extreme covariates	Yes	Yes	Yes	Yes
N	803,746	803,746	803,746	803,746
R^2^	0.067	0.063	0.090	0.085

The dependent variable is the logarithm of outages. CPP denotes coal power plants. The distance unit is 100 km. Standard errors are in the parentheses and are clustered at the county level. ∗*p* < 0.10, ∗∗*p* < 0.05, ∗∗∗*p* < 0.01.

In an alternative analysis, we conducted an alternative analysis after collecting province-level data on general grid reliability from the Compilation of Statistical Information on the Electric Power Industry. This reliability indicator, which spans from 0% to 100%, comprehensively assesses the condition of generation units, transformers, and transmission capacities. We categorized the data into two subgroups: those below and above the average of grid reliability. Our results (Table S6) indicate that regions with higher grid reliability exhibit smaller coefficients and are less susceptible to heatwave-induced outages.

Considering the reliance on various energy sources across regions, we have divided the data into two subgroups: regions with high renewable generation and those primarily dependent on fossil fuels. Our results (Table S7) show that regions with a significant share of renewable energy are more prone to heatwave-induced power outages than regions dependent on fossil fuels. This vulnerability is primarily due to the intermittent nature of renewable energy sources, which poses challenges until grid infrastructures are sufficiently upgraded to handle such fluctuations. This evidence aligns with literature that emphasizes the essential need for grid adaptations to facilitate the integration of renewables into energy systems.^24^^,^^42^^,^^43^

### Peak-hour outages

Due to the overlap of system loads with high temperatures, we would expect the probability of energy system disruptions increases during heatwaves.^10^^,^^52^ Given that peak electricity demand hours vary by province, we determined peak hours for each province based on their time-of-use electricity pricing regulations, which were compiled through a review of governmental releases and public news sources.^53^^,^^54^ Our results (Table 4) indicate that heatwaves lead to a 1.4% increase in peak-hour outages. This is expected since high electricity demand renders peak hours more susceptible to heatwaves. This finding aligns with previous studies,^10^^,^^23^ which indicate that high-temperature days challenge peak load management and grid operations. Additionally, we examined the disparate impacts on cities with varying levels of industrial production. Our results (Table S8) show that heatwave-induced outages are more prevalent in higher-industrial production cities. This suggests that industrial demands may exacerbate competition for electricity resources, thereby increasing the likelihood of outages during critical periods.

**Table 4:** Impacts of heatwaves on peak-hour outages

	Ratio of peak-hour outages	
	(1)	(2)
Heatwave	0.001 (0.002)	0.014∗∗∗ (0.001)
County-by-year fixed effects	Yes	Yes
Month fixed effects	Yes	Yes
Weather covariates	No	Yes
Climate extreme covariates	No	Yes
N	685,100	685,100

The dependent variable is the ratio of peak-hour outages. Standard errors are in the parentheses and are clustered at the county level. ∗*p* < 0.10, ∗∗*p* < 0.05, ∗∗∗*p* < 0.01.

### Projections under different scenarios

**Figure 3:**
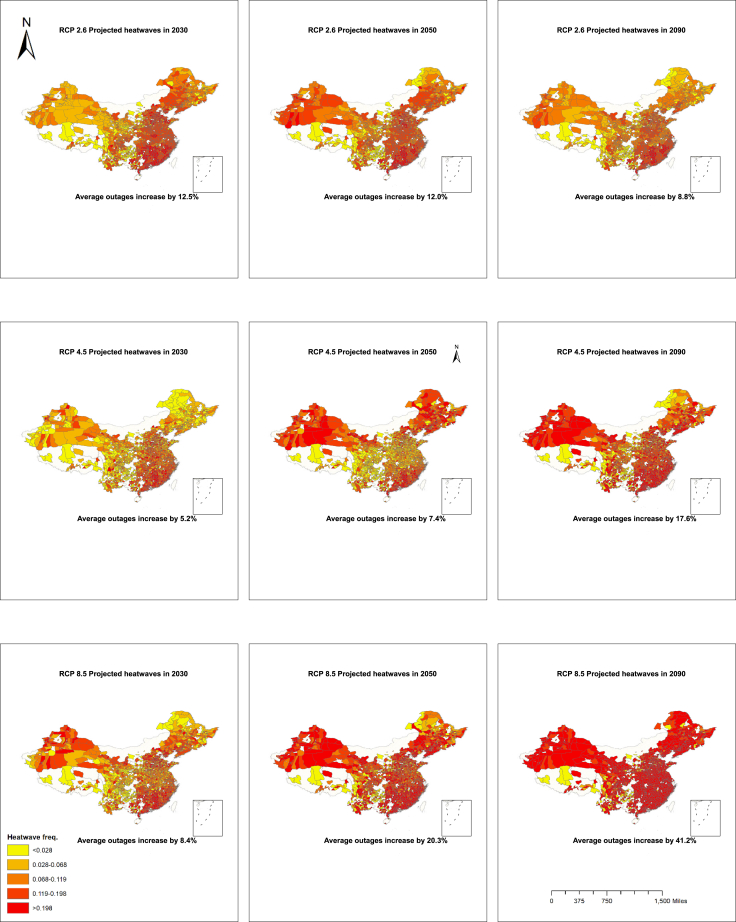
Distribution of heatwaves in scenarios of RCP2.6, RCP4.5, and RCP8.5

In this section, we use climate scenarios to project the future impacts of heatwaves on electricity supply reliability. We obtained the data from the Community Climate System Model (CCSM), supported by the National Center for Atmospheric Research (NCAR). Specifically, we employed monthly temperature projections under three different RCPs: RCP2.6, RCP4.5, and RCP8.5. We aligned the temperature projections with the geographical centers of cities using their latitudes and longitudes. We then calculated the projected changes in temperatures for 2030 (short-term), 2050 (middle-term), and 2090 (long-term) for each city since 2020. Figure 3 illustrates the distribution of heatwaves across these three scenarios. A greater frequency of heatwaves corresponds to a higher rate of outages. The average increase in outages is calculated based on the projected heatwaves and the heatwave-induced outage coefficients from Table 1, assuming that the mid-term and long-term effects would be similar to our estimations (Methods S2). As an alternative, higher-resolution climate projections are applied, which provide results generally consistent with the former results (Section S2, “Projections using alternative downscaled climate data”).^55^

Under the RCP2.6 scenario, the mean frequency of heatwaves will increase to a range of 0.093–0.113, compared to a baseline mean of 0.067 during our study period, which corresponds to an increase in outages of 8.8%–12.5%. Under the RCP4.5 scenario, heatwave-induced power outages are projected to increase by 5.2% in 2030, 7.4% in 2050, and 17.6% in 2090. In the absence of climate adaptation measures under RCP8.5, the long-term outage increase could reach up to 41.2%. The changes across different scenarios are not unidirectional due to temperature variability across latitudes and the differing hotspots.^56^^,^^57^ The magnitudes of these changes align with existing research, which suggests energy demand could increase by approximately 20% under moderate global warming and 25%–58% under severe warming.^15^^,^^58^ To gauge the economic impact of these outages, we estimated potential damages for each scenario, revealing that without improvements to grid infrastructure, annual damages could range from 9.7 to 23.3 billion yuan in 2030, 13.8 to 37.8 billion yuan in 2050, and 16.4 to 76.6 billion yuan in 2090 (Table 5).

**Table 5:** Projected outages under climate scenarios

		2030	2050	2090
RCP2.6	Average heatwave frequency	0.145	0.142	0.122
	Increase in outage occurrence	12.5%	12.0%	8.8%
	Economic losses (billion yuan)	23.3	22.3	16.4
RCP4.5	Average heatwave frequency	0.100	0.113	0.177
	Increase in outage occurrence	5.2%	7.4%	17.6%
	Economic losses (billion yuan)	9.7	13.8	32.7
RCP8.5	Average heatwave frequency	0.120	0.194	0.324
	Increase in outage occurrence	8.4%	20.3%	41.2%
	Economic losses (billion yuan)	15.6	37.8	76.6

Additionally, in Sections S3 and S4 “Future supply and demand projections” and “Future electricity sector scenarios,” we explore how future supply-demand mismatch (i.e., instances where supply fails to meet demand) and development of the electricity sector may impact heatwave-induced outages. We projected the increase in demand, supply, and supply-demand mismatch, respectively. Our findings indicate that power shortage (supply-demand mismatch) rates generally hover around 10% (Table 6). They differ from power outage rates since outages occur not only due to power shortages but also from failures in distribution and transmission infrastructure, such as malfunctioning substations and overloaded circuits. In the scenarios analysis, we built four stylized scenarios to explore further changes in power sector: reference, impacted renewable generation, low energy storage, and high energy storage. Our results indicate that as climate change affects renewable energy generation, the likelihood of power disruptions rises. Additionally, advancements in energy storage technology enhance the integration of renewable energy into the grid, improving the overall reliability of the power system.

**Table 6:** Projected increase in annual electricity demand, supply, and demand-supply mismatch due to climate change

	2030	2050	2090
**RCP2.6**			

Increased demand	0.56 trillion kWh	0.51 trillion kWh	0.58 trillion kWh
Increased supply	0.38 trillion kWh	0.73 trillion kWh	0.32 trillion kWh
Demand-supply mismatch	7.66%	8.55%	7.47%

**RCP4.5**			

Increased demand	0.45 trillion kWh	0.53 trillion kWh	0.74 trillion kWh
Increased supply	0.28 trillion kWh	1.09 trillion kWh	0.64 trillion kWh
Demand-supply mismatch	7.33%	9.03%	8.45%

**RCP8.5**			

Increased demand	0.50 trillion kWh	0.68 trillion kWh	1.22 trillion kWh
Increased supply	0.45 trillion kWh	1.36 trillion kWh	1.46 trillion kWh
Demand-supply mismatch	8.03%	9.98%	11.50%

### Robustness checks

#### Alternative definition of heatwaves

In this section, we use an alternative definition of heatwaves. Heatwaves can be defined by either relative or absolute thresholds. While the main context employs heatwaves defined by relative threshold (RHWs) based on the works of Xu et al. (2016)^30^ and Xu and Zhang (2022),^31^ this section utilizes alternative absolute threshold-defined heatwaves (AHWs). Specifically, a fixed value of 35°C is set as the threshold. Here, heatwave events comprise 0.96% of all observations. Our analysis (Table 7) is consistent with the main findings, indicating that heatwave events lead to more outages, although the magnitudes observed are larger than those reported earlier.

**Table 7:** Impact of heatwaves on outages

	Absolute threshold-defined heatwaves	Unplanned outages	Excluding other hazards
Heatwave	0.116∗∗∗ (0.006)	0.038∗∗∗ (0.002)	0.040∗∗∗ (0.002)
County-by-year fixed effects	Yes	Yes	Yes
Month fixed effects	Yes	Yes	Yes
Weather covariates	Yes	Yes	Yes
Climate extreme covariates	Yes	Yes	Yes
N	803,746	627,273	802,221
R^2^	0.084	0.089	0.090

The dependent variable is the logarithm of outages. Standard errors in parentheses are clustered at the county level. ∗*p* < 0.10, ∗∗*p* < 0.05, ∗∗∗*p* < 0.01.

#### Unplanned outages

In this section, we distinguish between planned and unplanned outages. Planned outages, which are pre-arranged, serve to maintain a reliable and safe operation of the power grid through routine equipment maintenance and necessary grid upgrades. Conversely, our focus here is specifically on the impact of heatwaves on unplanned or spontaneous outages. After excluding the planned outages, which constitute 22% of the total, we re-ran Equation 1. Our results (Table 7) remain statistically significant, indicating that heatwave-induced outages increase by 3.8%. This increase is consistent with the main results in Table 1.

#### Environmental hazards

Power outages during high-temperature events can be confounded by other environmental hazards, such as floods, snowstorms, thunderstorms, hail, earthquakes, and typhoons.^23^ To distinguish the outages caused by heatwaves from those by other natural hazards, we re-ran the analysis, excluding the days when other natural hazards occurred. We conducted an extensive search of natural hazards from the China Statistical Yearbook, the China Ministry of Emergency Management, governmental websites, and public news releases and reports.^59^^,^^60^ The search yielded in a dataset of 532 county-date level natural hazards during our study period. Our analysis reveals that, after excluding other natural hazards, heatwaves are associated with an increase of 4.0% in outages (Table 7), which is consistent with the main results in Table 1. Additionally, we ran a regression analysis of the number of household outages (in thousands) (Table S9). The results are significant and we found heatwave events lead to an increase of 1.5% per thousand household outages. However, due to limited and low-quality information on the number of affected households, this analysis is not included in the main analysis.

## Discussion

Climate change is expected to lead to a higher occurrence of heatwaves and subsequently result in increased power outages. Concurrent power outages and heatwaves would put human beings, especially vulnerable communities, at a higher risk of adverse effects. This study utilizes nationwide data on heatwaves and power outages in China to provide empirical evidence on the impact of heatwaves on electrical reliability. We found that heatwaves increase the frequency of outages by 3.9%–4.0% and increase the length of power outages by 7.9%–8.3%. Additionally, more intense and prolonged heatwaves contribute to an increased probability of power outages. In addition, we observed that the impacts of heatwaves on outages vary among different socio-demographics. Furthermore, we noted that outages during heatwaves increased with longer distances from power plants, and more outages occurred during peak hours than non-peak hours. Our projection results suggest that power supply reliability will face greater challenges in the future without adequate implementation of adaptive measures.

Our results have significant implications for similar countries and regions that are experiencing increasing temperatures and rapid growth in energy demand. Our results provide policymakers with valuable insights into the impact of climate change on power reliability and assist in accurate cost-benefit analyses. Furthermore, we have examined the heterogeneity across counties and across key socio-demographics. The heterogeneity analysis provides information about which vulnerable communities to prioritize for intervention and resource allocation. We aim to facilitate adaptative planning and implementation of policies to mitigate the impacts of climate change on energy systems. Furthermore, demand-side management, such as energy efficiency and human behavioral changes, are also recommended to increase building resilience to combat climate change.^19^^,^^61^

### Limitations of the study

There are limitations in our study. Firstly, the compound effects of heatwaves should be considered, as simultaneous and preconditioned weather and climate extremes often co-occur.^41^^,^^62^^,^^63^^,^^64^ Heatwaves may accompany high humidity, or conversely, with droughts. Moreover, compound hot extremes, where heatwaves persist both day and night,^65^^,^^66^ are likely to have a higher impact on electricity systems than estimates based solely daily mean temperature, as evidenced by their intensified health effects.^21^ Additionally, when heatwaves are preceded by events such as floods or tropical cyclones, the reliability, safety, and efficacy of electricity supply can be substantially compromised due to damage to key infrastructure.^67^^,^^68^ These factors should be incorporated into future assessments to provide a more comprehensive understanding of heatwave-induced outages.

Secondly, in the fixed-effects model, we assume constant treatment effects, which posit that the treatment effect remains the same across all counties and over time.^69^ However, the treatment effects can vary, and there may be a dynamic relationship between heatwaves and outages. To partially address this concern, we estimate the heterogeneous impacts among heatwave intensity and duration, peak hours and non-peak hours, and socio-demographics. To further explore the dynamic treatment effect, we used a one-day lag heatwave as one more robustness check (columns (3) and (4) in Table S10). We found that the lagged effects are present because of the faults in cables and joints cumulatively evolve toward power outages.^70^ Besides, precipitation may be correlated with relative humidity. We added a robustness check by removing relative humidity (Table S10).

Moreover, our analysis in the section “projections under different scenarios” does not completely capture the corresponding changes in power generation due to climate change. Under climate change, energy systems could become more vulnerable to extreme weather conditions, particularly with the integration of renewable energy.^24^^,^^42^ Simultaneously, energy demand is expected to increase due to climate change.^46^^,^^58^^,^^71^^,^^72^ We provide our estimation of these changes in Sections S3 and S4. As the estimation in the main text does not fully account for these aspects, it only provides a lower bound of the potential effects. We encourage interested readers to refer to valuable published studies,^52^^,^^72^^,^^73^ which provide more robust perspectives. Notably, extreme weather events have non-local impact on outages in other regions due to connected grid,^67^^,^^74^ which future work should take into account. Finally, this study captures the impacts of heatwaves without delving into the details of consumer behavioral changes. Heatwaves influence consumer energy behaviors in various ways such as driving higher cooling demand,^15^^,^^75^^,^^76^ influencing consumers’ adoption of cooling appliances,^46^ and also affecting investments in energy efficiency.^34^ Future studies are also needed in that aspect.

## Resource availability

### Lead contact

Further information and requests for resources and reagents should be directed to and will be fulfilled by the lead contact, Yueming (Lucy) Qiu (yqiu16@umd.edu).

### Materials availability

This study did not generate new unique reagents.

### Data and code availability

• Records of daily weather conditions were retrieved from the National Oceanic and Atmospheric Administration (NOAA) at https://www.ncei.noaa.gov/access/metadata/landing-page/bin/iso?id=gov.noaa.ncdc:C00516. The raw and processed electricity outage data were web-scraped from city government websites and can be assessed upon reasonable request. The socio-demographic data at the county level were collected from the China County Statistical Yearbook. Data on coal power plants were obtained from the Global Coal Plant Tracker at https://globalenergymonitor.org/projects/global-coal-plant-tracker/. Data were processed and analyzed in Stata (15.1) and R (4.3.1). The figures were produced in R studio and ArcMap (10.8.1).
• All custom code is available on GitHub from https://github.com/jingliang727/HW_outage.
• Any additional information required to reanalyze the data reported in this paper is available from the lead contact upon request.

## Acknowledgments

This work is supported by the 10.13039/501100001809National Natural Science Foundation of China (reference no. 72243001 and 72321002).

## Author contributions

All the authors conceived the research idea, designed the paper, and planned the analysis. J.L. wrote the initial draft of the paper and all the others also made large contribution to the revised draft.

## Declaration of interests

The authors declare no competing interests.

## STAR★Methods

### Key resources table

**Table:** 

REAGENT or RESOURCE	SOURCE	IDENTIFIER
**Deposited data**		

Climatic data	the National Oceanic and Atmospheric Administration (NOAA)	https://www.ncei.noaa.gov/access/metadata/landing-page/bin/iso?id=gov.noaa.ncdc:C00516
Electricity outages data	City government websites	Real-time web scraping
Socio-demographics data	China County Statistical Yearbook	https://www.stats.gov.cn/zs/tjwh/tjkw/tjzl/202302/t20230215_1908004.html
Coal power plants data	Global Coal Plant Tracker	https://globalenergymonitor.org/projects/global-coal-plant-tracker/.
Code for modeling, analysis and visualization used in the manuscript	This paper	https://github.com/jingliang727/HW_outage

**Software and algorithms**		

Stata 16	StataCorp LLC	https://www.stata.com/
R version 4.4.1 (2024-06-14)	RStudio, PBC	https://www.rstudio.com/

### Method details

#### Data

The climatic data used in this study were obtained from the National Oceanic and Atmospheric Administration (NOAA) and encompass weather records collected from 154 meteorological stations across China. These records include daily measurements of weather elements such as average temperature, sea level pressure, station pressure, visibility, wind speed, and precipitation.

We aligned the meteorological records with city centroids based on their latitudes and longitudes. We employed the inverse distance weighting approach,^34^^,^^35^^,^^36^ a method extensively used in the literature to impute weather and air pollution data.^35^^,^^36^^,^^77^^,^^78^^,^^79^ The inverse distance weighting method, widely recognized and employed in research for imputing weather and air pollution data from stations,^30^^,^^31^^,^^32^^,^^64^^,^^65^^,^^66^ was used to calculate the weighted average of climate records for cities.

The data on electricity outages in China were obtained through real-time web scraping from city government websites in real-time. The outage data includes detailed information on the location, start time, end time, length, and type (planned and unplanned) for each outage. We aggregated this data at the county-daily level. County-level cities, counties, districts in cities, and autonomous counties are treated as county-level administrative divisions. The total number of outage occurrences (and cumulative outage duration) in a county per day is calculated by summing the individual outage events (and their durations) across all locations within that county. To minimize the influence of extreme cold on outage data, we restricted our dataset to the summer months, from May to October. Outages lasting more than a week were excluded, as these are likely due to data updating errors. Outages may arise from various causes, including overloading, transformer failures, and aging infrastructure. Our analysis focuses on the net impact of heatwaves on increasing outage frequency, without distinguishing between the specific causes of outages. It should be noted that data reporting may be less comprehensive in some remote areas, which are often more prone to outages. Provinces such as Inner Mongolia, Xinjiang, Qinghai, and Hainan exhibited the highest percentages of missing data. Consequently, our estimates likely provide a conservative approximation of the actual effects.

In addition, since our outage data were obtained through web scraping, we have verified their consistency by comparing them with published Annual System Average Interruption Duration Index (SAIDI) and System Average Interruption Frequency Index (SAIFI) data (Table S11).^80^^,^^81^^,^^82^^,^^83^^,^^84^^,^^85^ To further ensure robustness, we conducted additional regression analyses using the annual SAIDI data at the city level (Table S12).

#### Empirical strategies

We employ a two-way fixed effects model (or a generalized difference-in-differences model) to investigate the impact of heatwave-induced electricity outages. We consider heatwaves as exogenous to outages, given that weather changes are naturally occurring and stochastic. Our empirical strategy exploits the variation across time and space, and the unexpected nature of heatwaves contributes to a robust research design. Our fixed effects models help to eliminate any county-specific time-invariant confounding factors, as well as some time-varying factors. Specifically, the following empirical model is applied:

(Equation 1) $$\mathrm{Ln}\left({Out\_frequency}_{id}\right)=\alpha_{i}+\beta_{1}{Heatwave}_{id}+X_{id}^{\prime }\theta +{\delta_{y}+\tau }_{m}+\varepsilon_{id}$$

(Equation 2) $$\mathrm{Ln}\left({Out\_length}_{id}\right)=\alpha_{i}+\beta_{1}{Heatwave}_{id}+X_{id}^{\prime }\theta +{\delta_{y}+\tau }_{m}+\varepsilon_{id}$$

where $\mathrm{Ln}({Out\_frequency}_{id}$ and $\mathrm{Ln}\left({Out\_length}_{id}\right)$ denote the logarithm of frequency (in incidents) and length (in hours) of power outages for county *i* at day *d,* respectively. The log transformation is taken to help achieve a normal distribution, as well as to facilitate the interpretation of our results as percentage changes in the dependent variable. Power outages can be planned, routinely scheduled, precautionary, or spontaneous; we categorize these into planned and unplanned outages. We include all types of outages and explore the overall effects, while also differentiating them in the robustness checks, with a particular focus on the unplanned ones.

${Heatwave}_{id}$ is equal to 1 if heatwaves are experienced and 0 otherwise. The covariates $X_{id}$ control for other climatic factors such as precipitation, wind, and humidity, as well as extreme climate events (e.g., floods, storms, and wildfires) and temporal cycles (e.g., holidays and weekends), to reduce the risk of omitted variable bias. The coefficient $\beta_{1}$ measures the effect of heatwaves on electricity outages. $\alpha_{i}$ represents the individual county-level fixed effects, controlling for time-invariant characteristics among counties, such as county energy infrastructure and demand-side management or policies. The year-fixed effects $\delta_{y}$ and month-of-year fixed effects $\tau_{m}$ capture time-varying variations among different years and months, such as the enforcement of energy policies and economic growth. They help to absorb the effects of unobservable homogenous shocks in the time dimension. The city-by-year fixed effects, when included also account for annual changes in each city such as policy shifts, economic and technological conditions, and infrastructure development. Since the sampling of our data is clustered at the county level, we cluster the standard errors at the county level.

The study further examines how heatwave intensity and duration affect power outages by incorporating temperature (in degrees Celsius), and duration of the heatwaves (in days), respectively. The models are specified as:

(Equation 3) $$\mathrm{Ln}\left({Out}_{id}\right)=\alpha_{i}+\beta_{2}{Heatwave\_intensity}_{id}+X_{id}^{\prime }\theta +{\delta_{y}+\tau }_{m}+\varepsilon_{id}$$

(Equation 4) $$\mathrm{Ln}\left({Out}_{id}\right)=\alpha_{i}+\beta_{3}{Heatwave\_Duration}_{id}+X_{id}^{\prime }\theta +{\delta_{y}+\tau }_{m}+\varepsilon_{id}$$

In the equations, $\mathrm{Ln}\left({Out}_{id}\right)$ denotes the logarithm of power outages for county *i* on day *d,* and has two measures: frequency and length. $\beta_{2}$ represents the marginal impacts of temperature changes during heatwaves (i.e., intensity) on the outages whereas $\beta_{3}$ indicates the impacts of heatwave duration on outages. ${Heatwave\_intensity}_{id}$ is temperature in degree Celsius conditional on it is a heatwave day. ${Heatwave\_Duration}_{id}$ is the duration of heatwaves in days. Other variables share the same definition as in Equation 1. The error terms are again clustered at the county level.

### Quantification and statistical analysis

We employ a two-way fixed effects model to study the impact of heatwaves on electricity outages. Our approach takes advantage of variations across both time and counties, allowing us to isolate the effect of heatwave-induced outages from other potential confounding factors. For the analysis, we use Stata 16 and R software, which provide tools for data cleaning, modeling, and visualization.

The statistical details of the statistical analysis, including the number of observations (counties) and the confidence levels used for confidence interval calculations, are outlined in the respective figure and table descriptions, legends and notes.
