# Supplementary material for: Impacts of heatwaves on electricity reliability: Evidence from power outage data in China
Source: iScience. 2025 Jan 21;28(2):111855. doi: 10.1016/j.isci.2025.111855 (PMC11848798; doi:10.1016/j.isci.2025.111855)
Supplement: Document S1. Figures S1–S4, Tables S1–S21, and Methods S1 and S2 [file mmc1.pdf]

iScience, Volume *28*

Supplemental information

Impacts of heatwaves on electricity reliability: Evidence from power outage data in China

Jing Liang, Yueming (Lucy) Qiu, Bo Wang, Xingchi Shen, and Shangwei Liu

Supplementary Information

Supplementary Figures

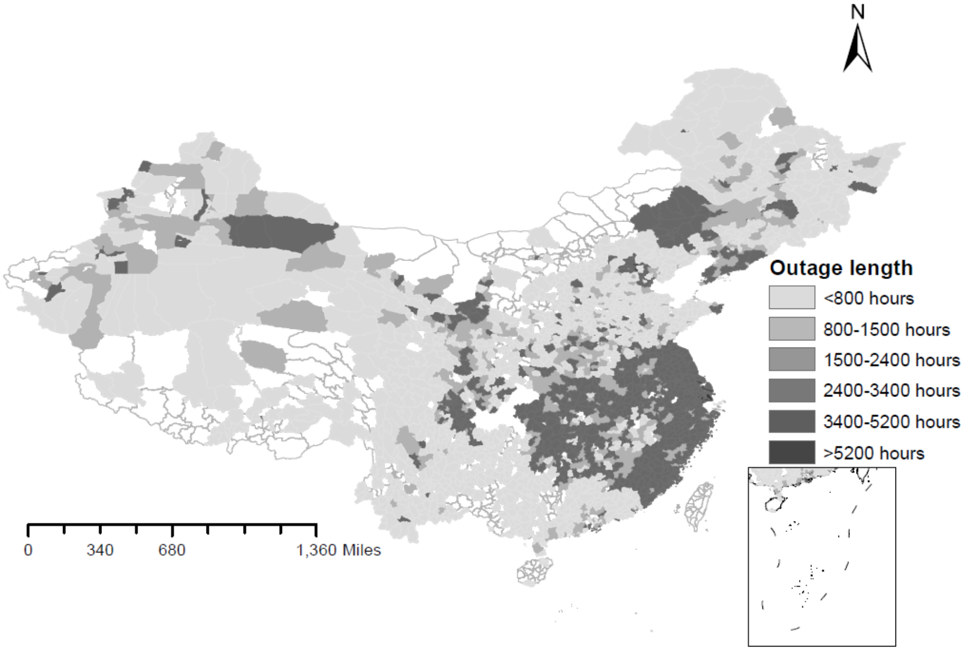

Figure S1. Duration (in hours) of residential outages during 2019–2021 in China

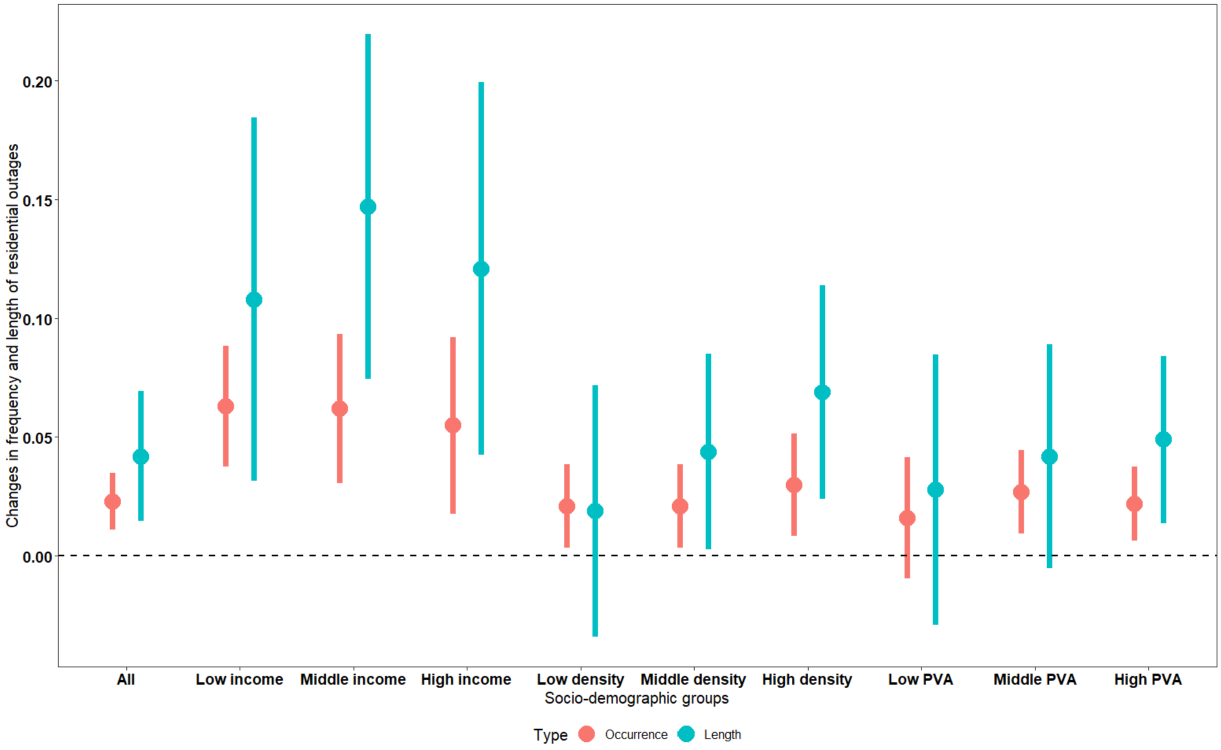

Figure S2. Heterogeneous impacts of heatwaves on power outages by different socio-demographic factors at the city level

Supplementary Tables

Table S1 Summary statistics

Variable	Obs.	Mean.	Std. dev.	Min.	Max.
Heatwave	803,746	0.094	0.292	0	1
Outages per county	803,746	2.463	1.410	0	104
Outage length (hours)	803,746	6.448	15.888	0	3,138
Distance-weighted daily temperature(°C)	803,746	24.815	3.504	11.204	32.329
Distance-weighted visibility (miles)	803,746	10.126	1.767	2.510	17.915
Distance-weighted wind speed (m/s)	803,746	2.432	0.460	0.607	9.055
Distance-weighted precipitation (inch/day)	803,746	0.208	0.187	0.001	7.626
Distance-weighted relative humidity	803,746	76.772	6.853	33.891	97.636
Economic loss from natural hazards (100 million yuan)	803,746	186.434	168.882	1.7	602.6
Wildfire	803,746	47.176	41.851	2	206
Holiday dummy	803,746	0.095	0.293	0	1

Notes: The summary statistics of outages refer to the average daily outages per county, which is the total of events across all locations. Distance-weighted variables are calculated as a weighted average using the inverse distance weighting approach, which aligns the data from meteorological stations with the county centroid based on their latitudes and longitudes.

Table S2 Impacts of heatwaves on residential power outages

	Outage occurrence	Outage length
Heatwave	0.040***	0.083***
	(0.005)	(0.011)
2019.year	0.000	0.000
	(.)	(.)
2020.year	0.016*	0.086***
	(0.010)	(0.020)
2021.year	0.066***	0.177***
	(0.011)	(0.023)
5.month	0.000	0.000
	(.)	(.)
6.month	0.117***	0.302***
	(0.010)	(0.025)
7.month	-0.011	-0.028
	(0.008)	(0.021)
8.month	-0.030***	-0.072***
	(0.008)	(0.023)
9.month	0.043***	0.116***
	(0.006)	(0.015)
10.month	0.080***	0.219***
	(0.008)	(0.021)
Weekend dummy	-0.134***	-0.321***
	(0.006)	(0.015)
Distance-weighted visibility	-0.001	-0.007**
	(0.001)	(0.003)
Distance-weighted wind speed (m/s)	0.021***	0.049***
	(0.003)	(0.007)
Distance-weighted precipitation (inch/day)	0.047***	0.087***
	(0.010)	(0.024)
Distance-weighted relative humidity	0.002***	0.005***
	(0.000)	(0.001)
Economic loss from natural hazards (in 100 million yuan)	0.069***	0.168***
	(0.022)	(0.044)
Wildfire dummy	0.002*	0.006**
	(0.001)	(0.003)
Holiday dummy	-0.231***	-0.613***
	(0.010)	(0.023)
_cons	-12.743***	-30.946***
	(4.004)	(8.019)
N	803746	803746
R^2	0.067	0.062

Notes: Month-fixed effects control for temporal variation using a set of indicators for month. County-by-year fixed effects control for the county-specific unobserved factors. Clustered at the city gives cluster-robust standard errors that reflect the clustering of data in a city. The dependent variable is the log of residential outages. Standard errors in the parentheses are clustered at the county level. * p<0.10, ** p<0.05, *** p<0.01.

Table S3 Power outage interruption costs and values of VoLL (the Value of Lost Load)

Country	Year	VoLL	Reference
Germany	2007	15.7 €/kWh	[1]
Republic of Ireland	2008	12.9 €/kWh	[2]
Cyprus	2009	6.5 €/kWh	[3]
Austria	2011	17.1 €/kWh	[4]
US	2012	$2.3/kWh	[5]
Notes: It also includes VoLL for Austria, New Zealand; Australia, Republic of Ireland			
Netherlands<br>US	2013	15.8 €/kWh	[6]
Notes: It also includes VoLL for 28 countries in the European Union			
China	2017	4.8–12.1 yuan/kWh	[7]
US	2018	$1.7–2.3 /kWh	[8]

Notes: Power outage interruption costs, or the Value of Lost Load (VoLL), represent the average cost to consumers per unit of unserved electricity due to outages. This encompasses both direct costs, such as spoiled food and lost productivity, and indirect costs, including inconvenience and potential health risks. This table includes only studies published after 2010. The most common methodologies employed are survey methods (i.e., willingness to pay (WTP) and willingness to accept (WTA)) and macroeconomic approaches. The mean and standard deviation of these costs were calculated after adjusting for inflation to the year 2020, and then converting to yuan.

Table S4 Projected residential outages under climate scenarios

		2030	2050	2090
RCP2.6	Average heatwave frequency	0.145	0.142	0.122
	Increase in outage occurrence	12.5%	12.0%	8.8%
	Economic losses (billion yuan)	9.7	3.2	3.1
RCP4.5	Average heatwave frequency	0.100	0.113	0.177
	Increase in outage occurrence	5.2%	7.4%	17.6%
	Economic losses (billion yuan)	1.3	1.9	4.5
RCP8.5	Average heatwave frequency	0.120	0.194	0.324
	Increase in outage occurrence	8.4%	20.3%	41.2%
	Economic losses (billion yuan)	2.2	5.2	10.6

Table S5 Marginal cost of grid reliability improvement

Country	Methods	Marginal cost	Reference
Pakistan	Econometrics method	0.023 Rupees per minute per customer (2018)	[9]
China	Distance function approach	59.66 to 74.65 yuan/per min per customer (2012–2018)	[10]
China	Distance function approach	$1.67 per hour per household (2017)	[11]
France	Distance function approach	10.7€ shadow price of quality (2003–2005)	[12]
UK	Econometrics method	30.7 pence per minute per customer (1995–2003)	[13]

Notes: We obtained the reported values in the literature, deflated them to 2020, and then transferred them to yuan. The mean is 16.2 yuan per minute per customer with a standard deviation of 33.2 yuan. The annual costs of upgrading a county's grid are calculated by multiplying the marginal cost per minute per customer by the number of customers and the average unserved electricity for the county.

Table S6 Impact of heatwaves and grid reliability on residential outages

	Low-reliability regions		High-reliability regions	
	Outage occurrence	Outage length	Outage occurrence	Outage length
Heatwave	0.055***	0.109***	0.029***	0.061***
	(0.004)	(0.009)	(0.003)	(0.008)
Month fixed effects	Yes	Yes	Yes	Yes
County-by-year fixed effects	Yes	Yes	Yes	Yes
Climate extreme covariates	Yes	Yes	Yes	Yes
N	404,260	404,260	399,486	399,486

Notes: The dependent variable is the log of residential outages. Standard errors in the parentheses are clustered at the county level. * p<0.10, ** p<0.05, *** p<0.01.

Table S7 Impact of heatwaves on residential outages in different regions

	Regions with high fossil generation		Regions with high renewable generation	
	Outage occurrence	Outage length	Outage occurrence	Outage length
Heatwave	0.033***	0.072***	0.082***	0.143***
	(0.002)	(0.006)	(0.007)	(0.016)
County-by-year fixed effects	Yes	Yes	Yes	Yes
Month fixed effects	Yes	Yes	Yes	Yes

Weather covariates	Yes	Yes	Yes	Yes
Climate extreme covariates	Yes	Yes	Yes	Yes
N	618,718	618,718	185,028	185,028

Notes: The dependent variable is the log of residential outages. Regions with high renewable generation (wind, solar, and hydroelectric) refer to the top five provinces, namely, Sichuan, Yunnan, Hubei, Inner Mongolia, and Guizhou provinces. Standard errors in the parentheses are clustered at the county level. * p<0.10, ** p<0.05, *** p<0.01.

Table S8 Impacts of heatwaves on residential peak-hour power outages in cities of different industrial production levels

	Light-industry cities		Middle-industry cities		Heavy-industry cities	
	Outage occurrence	Outage length	Outage occurrence	Outage length	Outage occurrence	Outage length
Heatwave	0.020***	0.034***	0.030***	0.058***	0.035***	0.076***
	(0.003)	(0.008)	(0.003)	(0.009)	(0.004)	(0.009)
County-by-year fixed effects	Yes	Yes	Yes	Yes	Yes	Yes
Month fixed effects	Yes	Yes	Yes	Yes	Yes	Yes
Weather covariates	Yes	Yes	Yes	Yes	Yes	Yes
Climate extreme covariates	Yes	Yes	Yes	Yes	Yes	Yes

Notes: Month-fixed effects control for temporal variation using a set of indicators for months. County-by-year fixed effects control for the county-specific unobserved factors across different years. Clustered at the city give cluster-robust standard errors that reflect the clustering of data in a city. The cities are divided into three equal groups based on their percentage of secondary industry production to total gross production[14–16]. The dependent variable is the log of residential outages. Standard errors in the parentheses are clustered at the county level. * p<0.10, ** p<0.05, *** p<0.01.

Table S9 Impact of heatwaves on the number of household outages

	Outage occurrence	
Heatwave	0.015***	0.015***
	(0.005)	(0.005)
County fixed effects	Yes	No
Year fixed effects	Yes	No
Month fixed effects	Yes	Yes
Weather covariates	Yes	Yes
Climate extreme covariates	Yes	Yes
County-by-year fixed effects	No	Yes
N	25,251	25,251

Notes: The dependent variable is the log of residential outages in a thousand households. * p <0.10, ** p<0.05, *** p<0.01. Standard errors in the parentheses are clustered at the county level.

Table S10 Impacts of heatwaves on residential power outages

	Outage occurrence	Outage length	Outage occurrence	Outage length
Heatwave	0.039***	0.079***		
	(0.002)	(0.006)		
Lag of heatwave			0.040***	0.087***
			(0.002)	(0.006)
County-by-year fixed effects	Yes	Yes	Yes	Yes
Month fixed effects	Yes	Yes	Yes	Yes
Weather covariates	Yes	Yes	Yes	Yes
Excluding relative humidity	Yes	Yes	No	No
Climate extreme covariates	Yes	Yes	Yes	Yes
N	803,746	803,746	801,024	801,024

Notes: The dependent variable is the log of residential outages. Standard errors in the parentheses are clustered at the county level. * p<0.10, ** p<0.05, *** p<0.01.

Table S11 Average SAIDI and SAIFI in China

	2018	2019	2020	2021
Average SAIDI	15.26	13.72	11.87	11.26
Urban	4.72	4.50	4.82	4.89
Rural	18.95	17.03	14.51	14.06
Average SAIFI	3.18	2.99	2.69	2.77
Urban	1.23	1.08	1.17	1.24
Rural	3.99	3.67	3.25	3.45

Table S12 Impacts of heatwaves on residential power outages

	(1)	(2)
	SAIDI	SAIDI
Heatwave	0.00109	0.000187
	(0.00301)	(0.00326)
2020.year	0.0348	0.00854
	(0.0338)	(0.0626)
2021.year	0.229***	0.156**
	(0.0461)	(0.0698)
City fixed effects	Yes	Yes
Year fixed effects	Yes	Yes

Weather covariates	Yes	Yes
Climate extreme covariates	No	Yes
Constant	2.308***	5.569**
	(0.100)	(2.509)
Observations	803	803
R^2	0.121	0.134
No. of cities	274	274

Notes: The dependent variable is the log of the annual SAIDI. Standard errors in parentheses are clustered at the city level. * p<0.10, ** p<0.05, *** p<0.01. The city-level SAIDI is replaced by utility-level reported data when missing. The reason for the difference in regression coefficients and their statistical significance might be that aggregated SADI data at the city-year level fails to capture some micro-level patterns than analyses at the county-daily level. Thus, it hides some data variation at a more granular level due to aggregation bias[17].

Supplementary Note

Methods S1: Justification of approaches, related to STAR Methods.

We acknowledge the great contribution made by other approaches and are fully aware of the excellent works under the physics-informed approach (e.g.,[18–20]). However, our econometrics analysis has the following advantages. (1) Our method can disentangle the effect of heatwaves on power outages, which is very challenging, if feasible, for engineering models. The factors causing outages are very complex and diverse, many of which are not solely related to physical systems. The econometrics analysis isolates the effect of heatwaves from all other influencing factors. It considers not only the physical process but also accounts for the influences from diverse non-physical factors such as human preventative and adaptive behaviors. (2) Our analyses are conducted at a very granular county level. Given that not all data at the county level are available for engineering modeling (such as transformer and distribution substation data at the county level), it is almost infeasible to model the power outages at the local level currently. The empirical estimation from econometric analyses could also have the potential to be fed into systems engineering and simulation studies. Given that there is no study to disentangle the impact of heatwaves on power outages yet, we take advantage of the historical data and address such a gap.

Therefore, the modeling approach to study outages should include coupled models such as the weather model, distribution network model, and response model, which can be an important area for future research. Also note that although systematic modeling is feasible, accumulative errors with inappropriate assumptions at all stages threaten to undermine accuracy.

Methods S2: Calculation of projections, related to Figure 3.

The projected future heatwave frequency is estimated based on the escalation of temperature. The heatwave and high-temperature threshold are defined in the same way in the main text (i.e., the 99th percentile). The projected temperatures in a city in different scenarios are projected according to temperature changes from the CCSM. New heatwave frequency is thus the heatwave days divided by all days in a year. The increases in heatwave-induced outages in different future scenarios are estimated based on the scaling of the heatwave-induced outages shown in Table 1. We assume for each county, the average impacts estimated in our main text still hold, which is one more heatwave leads to a 3.9% increase in outages. Nationally (around 3000 counties), as the total

heatwave occurrences across all counties increase by a certain percentage, we assume the national outages will increase proportionally. The economic losses caused by more outages are estimated the same way the back-of-the-envelope analysis in the section "Heatwave-induced outages".

Section S1 Failure rates for electric components

Reports indicate that in 2020, the failure rate for overhead lines was 10.35 per 100 km/year, 4.06 per 100 km/year for cable lines, 0.28 per 100 units/year for transformers, and 0.34 per 100 units/year for circuit breakers[21]. The failure indicators for 13 types of electric components are summarized in the following table (Table S21)[22]. In 2021, the failure rate for overhead lines was 9.84 per 100 km/year, 3.96 per 100 km/year for cable lines, 0.40 per 100 units/year for transformers, and 0.50 per 100 units/year for circuit breakers[22]. In 2022, these rates decreased significantly: 3.85 per 100 km/year for overhead lines, 1.43 per 100 km/year for cable lines, 0.17 per 100 units/year for transformers, and 0.15 per 100 units/year for circuit breakers[23].

We also examined the correlation between heatwaves and failures of specific electric components. The correlation coefficient between the national average temperature and cable line faults is 0.39, 0.79 for transformers, and 0.99 for circuit breakers. This suggests that heatwaves increase the failure rates of electric components, leading to power outages. As detailed nationwide data on component failures is not yet available, the above correlation analysis is preliminary.

Table S13 Reliability indicator of electric components for transmission lines at 220 kV and above in China (2020)

Facility Type	Facility/ Line Length (km)	Forced Outage Rate (%)	Availability (%)	Unplanned Outage Occurrences	Unplanned Outage Duration	Planned Outage Occurrences	Planned Outage Duration
Overhead Line	8387.6	0.046	99.466	436	0.17	4698	44.029
Transformer	21696.0	0.197	99.63	76	0.342	5099	31.715
Coupling Capacitor	7139.0	0.013	99.989	1	0.0	96	0.937
Wave Trap	12173.0	0.015	99.982	2	0.002	192	1.382
Cable Line	72.8	0.029	99.970	3	0.025	81	2.59
Reactor	4520.0	0.406	99.761	20	0.69	533	20.007
Circuit Breaker	52460.0	0.174	99.839	122	0.042	7623	13.957
Current Transformer	150409.0	0.008	99.948	40	0.011	7965	4.511
Combined Apparatus	9616.0	0.024	99.955	88	0.014	10206	3.552
Busbar	14240.0	0.148	99.947	22	0.384	816	4.211
Voltage Transformer	98443.0	0.016	99.945	21	0.002	5707	4.803
Disconnector Switch	185111.0	0.007	99.965	32	0.002	6001	3.078
Surge Arrester	167085.0	0.012	99.953	26	0.003	6939	4.145

Note: Total Facility/Line Length (km): for overhead lines and cable lines, the unit is per 100 kilometers; for other equipment, the unit is in sets or sections. Planned Outage Duration (times): for overhead lines, the unit is hours per 100 kilometers per year; for other equipment, the unit is hours per unit (sets, sections) per year.

Section S2 Projections using alternative downscaled climate data

We also applied available downscaled data from NASA's Earth Exchange Global Daily Downscaled Projections (NEX-GDDP)[24,25]. We accessed the NEX-GDDP-CMIP6 data and obtained daily average temperature data at a higher resolution (0.25 degrees). The results under RCP2.6 and RCP4.5 (Figure S3) align with previous findings, while those for RCP5.8 are substantially higher, likely due to the underestimation of extreme events at lower resolutions.

Table S14 Projected outages under climate scenarios

		2030	2050	2090
**RCP2.6**	Average heatwave frequency	0.14	0.179	0.207
	Increase in outage occurrence	13.9%	21.3%	26.6%
	Economic losses (billion yuan)	26.0	33.3	38.5
**RCP4.5**	Average heatwave frequency	0.137	0.186	0.246
	Increase in outage occurrence	13.3%	22.6%	34.0%
	Economic losses (billion yuan)	25.5	34.6	45.8
**RCP8.5**	Average heatwave frequency	0.648	0.654	0.664
	Increase in outage occurrence	110.4%	111.5%	113.4%
	Economic losses (billion yuan)	120.5	121.7	123.5

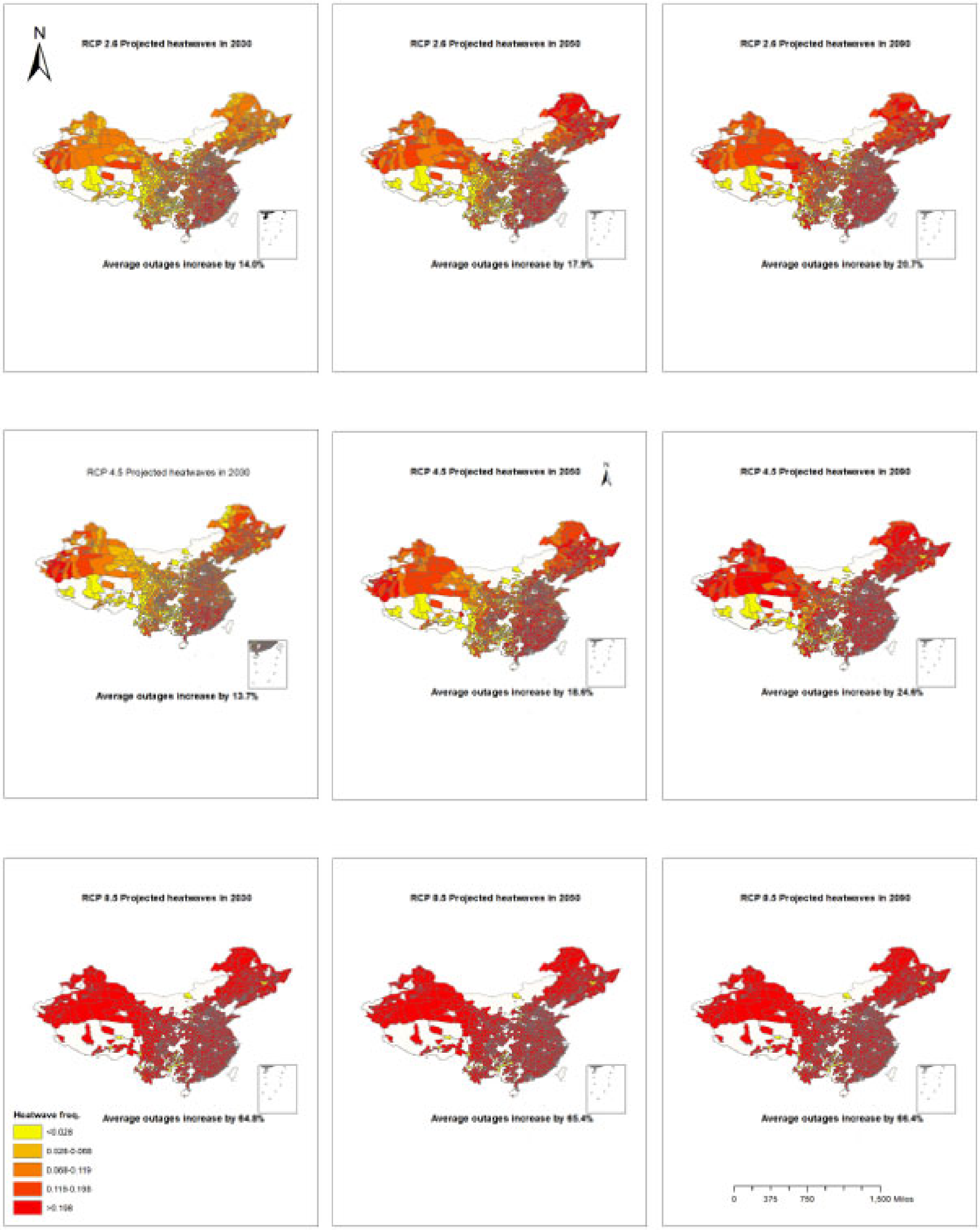

Figure S3. Distribution of heatwaves in scenarios of RCP2.6, RCP4.5, and RCP8.5 using NEX-GDDP-CMIP6 data

Section S3 Future demand and supply projections
Demand

Our analysis focuses on the relative changes in temperature-responsive cooling demand induced by heatwaves (Table S15). The electricity demand increment is calculated as $\Delta D_{i,d}$=$D\_base_{i,d} \times \beta_{i,d} \times \Delta t_{i,d}$. There are three critical parameters in the estimation: (1) the base consumption $D\_base_{i,d}$; (2) the sensitivity of demand changes to heating $\beta_{i,d}$, which is the percentage increment of demand relative to $D\_base_{i,d}$ per degree; (3) the temperature changes $\Delta t_{i,d}$. The temperature response parameters $\beta_{i,d}$ are sourced from existing studies including empirical temperature-response curves[26–28]. They are assumed to be 3% per °C for non-hot (Northern) regions, and 5% per °C for hot (Southern) regions when the temperature is 22–25°C. For temperatures above 25°C, the sensitivity increases to 8% per °C for non-hot regions and 10% per °C for hot regions. The base level of annual electricity consumption, $D\_base_{i,d}$, is obtained from the China City Statistical Yearbook[29]. We then disaggregate annual consumption to the daily level, factoring in daily variability representative of the load for provinces where the cities are located[30]. $\Delta t_{i,d}$ is based on the projected temperature from the Community Climate System Model (CCSM). The annual increase in cooling demand across China represents the sum of daily increases in electricity consumption due to temperature rises for all locations.

Table S15 Increase in annual electricity demand due to increased high-temperature extremes

	2030	2050	2090
RCP2.6	0.56 trillion kWh	0.51 trillion kWh	0.58 trillion kWh
RCP4.5	0.45 trillion kWh	0.53 trillion kWh	0.74 trillion kWh
RCP8.5	0.50 trillion kWh	0.68 trillion kWh	1.22 trillion kWh

Supply

The future supply is projected in a similar fashion. We focus on the vulnerability of different energy sources of electricity generation to high temperatures during heatwaves. The electricity supply decrement for province i at day d is $\Delta S_{i,d}$=$\sum_j^n S\_base_{i,j,d} \times \gamma_{j,d} \times \Delta t_{i,d}$. There are three critical parameters: (1) the projected base supply $S\_base_{i,j,d}$ for province i for energy source j at day *d*; (2) the vulnerability of different energy sources to high temperatures $\gamma_{j,d}$, which indicates the percentage decrease of supply relative to $S\_base_{i,j,d}$ per degree; (3) the temperature changes $\Delta t_{i,d}$. $\gamma_{j,d}$, which quantifies how susceptible each energy type, is derived from existing literature that discusses the electrical and thermal performance characteristics of different technologies (Table S16). $\Delta t_{i,d}$ is the same as the former. The base supply S_base is estimated through the following steps. (1) National supply prediction by fuel type: using the GCAM model, we predict national supply by fuel type under three SSP-RCP scenarios. The total supply in 2030, 2050, and 2090 is estimated as 12.00, 16.30, and 12.05 trillion kWh. (2) Disaggregation to provincial levels. National supply is disaggregated into provincial levels based on the provincial energy mix from [[31]]; (3) Daily disaggregation at the provincial level. For each province, its supply at the annual level is further disaggregated into the daily level by imposing the intra-annual variation. The daily wind and solar variation are based on the daily potentials from [[32]]. Daily variability of nuclear, coal, natural gas, and hydro generation is obtained from public reports and government releases[29,33].

The decrement in generation is displayed in Table S17. We find that high-temperature extremes reduce the average generation by 2.3–3.7% for RCP 2.6, 4.5 and 8.5 in 2030, 4.5–8.3% by 2050, and 2.7–12.1% by 2090 (the percentage is calculated as the decrement in generation due to vulnerability to high temperature divided by GCAM predicted supply). This is consistent with existing studies, which show that the electricity losses are 4.4–19% in the mid-term (e.g., [29],[30]).

Table S16 Parameters on the vulnerability of electricity generation to high temperatures

Type	Parameters	Explanation	References
Coal	0.1% /°C	One degree increase in temperature leads to an efficiency reduction of 0.09% for coal-fired power plants	[35]
		Each degree increase in temperature reduces the efficiency of thermal electric plants by 0.12-0.45%	[36]
Natural gas	0.1%/°C	Natural gas combustion turbines have an efficiency loss of 0.1%°C above ideal operating conditions;	[37]
		Temperature increase reduces average generating capacity by 2-3% for RCP 4.5-8.5 by the 2060s	[35]
Hydro	3.0%	Hydropower potential will change by -2.2 to -5.4% for 2020–2050 and 2070–2099, respectively	[38]
Nuclear	0.3%/°C below 30°C; 1.1%/°C above 30°C	One degree increase in the coolant temperature causes a decrease in power output and efficiency of 0.39% and 0.16%, respectively, in a nuclear power plant	[37]
		One degree increase for ambient temperatures between -7°C and 20°C reduces output by 0.3–0.4%; output decreases by 0.96–1.10% per degree over 20°C	[37]
		Load reduction reaches 11.8% if river temperature increases by 5 degrees	[39]
Solar PV	0.7%/°C	High temperatures lead to a drop in the maximum capacity and conversion efficiency of 0.66%/°C and 0.08%/°C, respectively	[37]
		Electricity conversion efficiency decreases by about 3–6‰ per degree	[40]
		Per degree increase in the cell temperature leads to an efficiency loss between 0.4% and 0.5%	[41]
Wind	0.1%	Annual wind power generation decreases by around 0.1% of their average production due to climate change	[42]
		Wind generation potential will decrease (3–4%) under RCP 4.5 and RCP8.5	[43]

Table S17 Increase in annual electricity supply

	2030	2050	2090
RCP2.6	0.38 trillion kWh	0.73 trillion kWh	0.32 trillion kWh
RCP4.5	0.28 trillion kWh	1.09 trillion kWh	0.64 trillion kWh
RCP8.5	0.45 trillion kWh	1.36 trillion kWh	1.46 trillion kWh

Supply-demand mismatch (power shortage)

Finally, we integrate the projected demand with the projected supply to assess the supply-demand mismatch or power shortage rate. This rate is calculated at the province-day level, aligning with the granularity of the projected demand data. Consequently, city-level supply projections are aggregated to the province level to facilitate this analysis. The national outage rate is estimated using the following equation:

$$Out_{i,d} = \begin{cases} 1 \; if \; ED_{i,d} > ES_{i,d} \\ 0 \quad otherwise \end{cases} \quad \text{(A1)}$$

$$PS^N = \frac{\sum_1^N Out_{id}}{Nd} \quad \text{(A2)}$$

Where the dummy variable $Out_{i,d}$ indicates whether demand exceeds supply in province *i* on day *d*. $ED_{i,d}$ denotes the demand and $ES_{i,d}$ is the supply. PS^N represents the national power shortage rate, which is calculated as the ratio of cumulative days during which demand exceeds supply across all provinces and days. *Nd* represents the total number of observations, which is the product of the number of provinces and the number of days observed for each province.

The results in Table S18 show that power outage rates generally hover around 10%. A comparison with Table 5 reveals similarities in some scenarios but differences in others. This variance stems from the fact that power shortage rates only assess the imbalance between supply and demand, without considering failed distribution, substation, and local transformers.

Table S18 Power shortage rate

	2030	2050	2090
RCP2.6	7.66%	8.55%	7.47%
RCP4.5	7.33%	9.03%	8.45%
RCP8.5	8.03%	9.98%	11.5%

We have calculated the additional power plant capacity required to address potential demand surges during heatwaves. This estimation is based on our previous calculations of electricity demand. The national capacity ΔC is estimated by $\Delta C = \sum_g \max_d D_{g,d} \times \partial_g$, where $D_{g,d}$ represents the daily electricity demand for region *g*. Considering that regions are typically interconnected and the supply network operates at a regional level, we have included eight regions as outlined by [32].

The term $\max_{d} D_{g,d}$ represents the peak of demand across all days for each region. The factor ∂_g is the proportion of single highest peak-hour demand relative to the total daily demand, calculated based on the daily demand profiles reported by the NDRC[30] . ΔC represents the cumulative peak-hour demand across all regions. As shown in Table S19, to mitigate the impacts of heatwaves, it is estimated that between 220 and 620 power plants, each with a capacity of 1,000 MW per hour, need to be constructed (in reality, the type of energy sources and storage capacity should also be considered). These additional plants would primarily function as peaking power plants, operational only during periods of high demand[44]. Note this high demand could also be met by increasing the utilization rate of existing power plants, especially since China's current capacity is underutilized, operating below 50%[45]. Coal power plants, in particular, could serve as peaking power plants in China, meeting reliability requirements by ramping up and down more efficiently. Furthermore, high demand might also be addressed through storage technologies or end-user services, potentially obviating the need for additional power sector infrastructure[46].

Table S19 Additional capacity needed due to increased temperature

	2030	2050	2090
RCP2.6	0.22 billion kW	0.21 billion kW	0.25 billion kW
RCP4.5	1.27 billion kW	2.73 billion kW	2.22 billion kW
RCP8.5	2.90 billion kW	2.89 billion kW	6.20 billion kW

Note: An increased peak demand of 0.22 billion kW indicates around 220 additional power plants with 1,000 MW needed to be built by 2030.

Besides, we are fully aware that our projection of future supply and demand is a simplified attempt in our efforts to assess power outages during heatwaves. Many valuable published studies, although not focusing on power outages as we do, have examined energy consumption and supply under climate change. Interested readers are directed to these papers listed in Table S20.

Table S20 Examples of studies on future energy projections

	References
Change in energy demand impacted by climate change/high temperature	e.g., [42],[43]
Change in energy supply impacted by climate change/high temperature	e.g., [31,35,49]
Supply-demand match/ power shortage	e.g., [26]; [32]

Section S4 Future electricity sector scenarios

We explore how the potential changes in the electricity sector may impact heatwave-induced power outages by constructing electricity sector scenarios. We construct the following scenarios: (1) the Reference Scenario, where the current energy mix remains unchanged; (2) the Impacted Generation Scenario, where electricity generation from fossil and non-fossil sources is impacted differently by climate change; (3) the Battery Storage Deployment Scenario, where battery technology mitigates the variability of renewable generation in the electricity grid, in addition to the impacts outlined in scenarios (2). Note that these scenarios are highly stylized and are intended solely to explore comparative changes in the electricity sector. Altogether, we have developed four scenarios: Reference, Impacted Generation, Low Energy Storage, and High Energy Storage.

Table S21 Stylized future electricity sector scenarios

Scenarios	2030	2050
Reference	No change (same as 2020)	No change (same as 2020)
Impacted Generation	Generation from both fossil and renewable sources (increase by 50%) is compromised by climate change	Generation from both fossil and renewable sources (increase by 100%) is compromised by climate change
Energy Storage	Battery storage deployment increases by 50% (Low), by 100% (High)	

The Reference scenario adopts the temperature projection in RCP2.6. In the Impacted Generation scenario, it is assumed that renewable energy, projected to increase by 50% in 2030 and 100% in 2050, will be impacted by climate change. The impact factors are listed in Table S21. In the Energy Storage scenario, we assume that the low and high energy storage scenarios correspond to 50% (50% storage) and 20% (100% storage) of renewable generation being affected by climate change, respectively. It is important to highlight that a reduction in generation does not necessarily translate into increased outages. This is because system dispatching and demand response can mitigate some of the negative impacts, although these factors are not fully incorporated into our simplified model. As such, our estimates should be considered as upper bounds on the potential effects.

Our results (Figure S4) indicate that increased renewable energy generation (Impacted Generation Scenario) contributes to a higher probability of power disruptions, increasing by 0.62% in 2030 and by 0.79% in 2050. Conversely, the deployment of additional energy storage (High Energy Storage Scenario) facilitates greater integration of renewable energy and enhances the overall system reliability. Here, the projected increase in the likelihood of power disruptions is modest, at 0.16% in 2030 and 0.17% in 2050. These results align with existing research which indicates that as more renewable generation is incorporated into the grid, power reliability and stability may be increasingly compromised by extreme weather conditions[26,50,51].

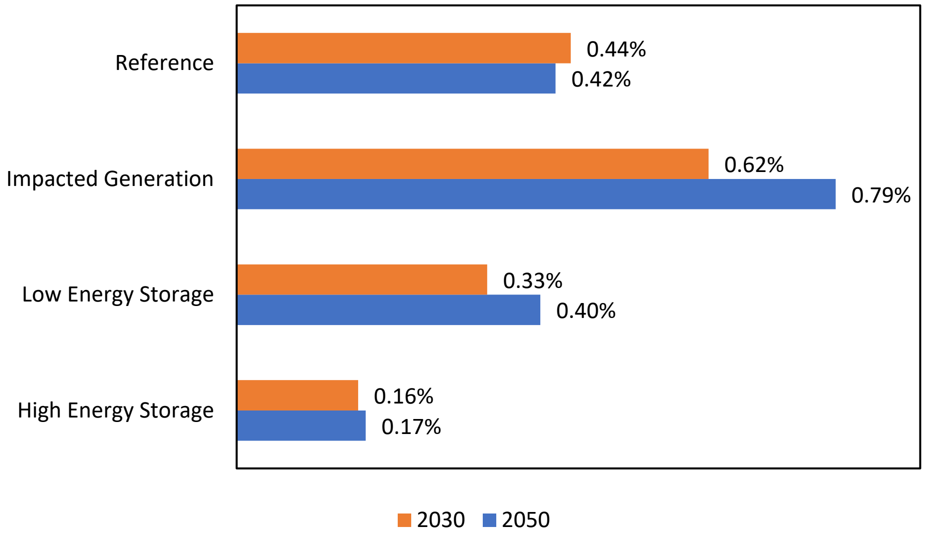

Figure S4. Impacts of future energy system on the probability of power outages in four scenarios

Appendix references

1. Praktiknjo, A.J., Hähnel, A., and Erdmann, G. (2011). Assessing energy supply security: Outage costs in private households. Energy Policy *39*, 7825–7833. https://doi.org/10.1016/j.enpol.2011.09.028.
2. Leahy, E., and Tol, R.S.J. (2011). An estimate of the value of lost load for Ireland. Energy Policy *39*, 1514–1520. https://doi.org/10.1016/j.enpol.2010.12.025.
3. Zachariadis, T., and Poullikkas, A. (2012). The costs of power outages: A case study from Cyprus. Energy Policy *51*, 630–641. https://doi.org/10.1016/j.enpol.2012.09.015.
4. Reichl, J., Schmidthaler, M., and Schneider, F. (2013). The value of supply security: The costs of power outages to Austrian households, firms and the public sector. Energy Economics *36*, 256–261. https://doi.org/10.1016/j.eneco.2012.08.044.
5. London Economics International LLC, "Estimating the Value of Lost Load," Electric Reliability Council of Texas, Inc. (ERCOT), 2013
6. Shivakumar, A., Welsch, M., Taliotis, C., Jakšić, D., Baričević, T., Howells, M., Gupta, S., and Rogner, H. (2017). Valuing blackouts and lost leisure: Estimating electricity interruption costs for households across the European Union. Energy Res. Soc. Sci. 34, 39–48. https://doi.org/10.1016/j.erss.2017.05.010.
7. Chen, H., Chen, X., Niu, J., Xiang, M., He, W., and Küfeoğlu, S. (2021). Estimating the marginal cost of reducing power outage durations in China: A parametric distance function approach. Energy Policy *155*, 112366. https://doi.org/10.1016/j.enpol.2021.112366.
8. Baik, S., Davis, A.L., Park, J.W., Sirinterlikci, S., and Morgan, M.G. (2020). Estimating what US residential customers are willing to pay for resilience to large electricity outages of long duration. Nat Energy *5*, 250–258. https://doi.org/10.1038/s41560-020-0581-1.
9. Mirza, F.M., and Mushtaq, I. (2022). Estimating the marginal cost of improving services quality in electricity distribution utilities of Pakistan. Energy Policy *167*, 113061. https://doi.org/10.1016/j.enpol.2022.113061.
10. Yuan, P., Pu, Y., and Liu, C. (2021). Improving electricity supply reliability in China: Cost and incentive regulation. Energy *237*, 121558. https://doi.org/10.1016/j.energy.2021.121558.
11. Chen, H., Chen, X., Niu, J., Xiang, M., He, W., and Küfeoğlu, S. (2021). Estimating the marginal cost of reducing power outage durations in China: A parametric distance function approach. Energy Policy *155*, 112366. https://doi.org/10.1016/j.enpol.2021.112366.
12. Coelli, T.J., Gautier, A., Perelman, S., and Saplacan-Pop, R. (2013). Estimating the cost of improving quality in electricity distribution: A parametric distance function approach. Energy Policy *53*, 287–297. https://doi.org/10.1016/j.enpol.2012.10.060.
13. Jamasb, T., Orea, L., and Pollitt, M. (2012). Estimating the marginal cost of quality improvements: The case of the UK electricity distribution companies. Energy Econ. *34*, 1498–1506. https://doi.org/10.1016/j.eneco.2012.06.022.
14. National Bureau of Statistics. *China City Statistical Yearbook.* China Statistics Press, 2020.
15. National Bureau of Statistics. *China City Statistical Yearbook.* China Statistics Press, 2021.

16. National Bureau of Statistics. *China City Statistical Yearbook.* China Statistics Press, 2022.
17. Garrett, T.A. (2003). Aggregated versus disaggregated data in regression analysis: implications for inference. Economics Letters *81*, 61–65. https://doi.org/10.1016/S0165-1765(03)00149-6.
18. Wang, Z., Hong, T., and Li, H. (2021). Informing the planning of rotating power outages in heat waves through data analytics of connected smart thermostats for residential buildings. Environ. Res. Lett. *16*, 074003. https://doi.org/10.1088/1748-9326/ac092f.
19. Sheng, M., Reiner, M., Sun, K., and Hong, T. (2023). Assessing thermal resilience of an assisted living facility during heat waves and cold snaps with power outages. Building and Environment *230*, 110001. https://doi.org/10.1016/j.buildenv.2023.110001.
20. Lin, N., & Xi, D. (2023). Likelihood of back-to-back tropical cyclone hazards is increasing. *Nature Climate Change*, *13*(3), 220-221.
21. China Electricity Administration (CEA). National Electricity Reliability Annual Report. 2020. https://prpq.nea.gov.cn/uploads/file1/20211009/616107fe94a8e.pdf
22. China Electricity Council (CEA). National Electricity Reliability Annual Report. 2021. http://prpq.nea.gov.cn/uploads/file1/20230308/640801ab6e248.pdf
23. China Electricity Council (CEA). National Electricity Reliability Annual Report. 2022. http://prpq.nea.gov.cn/uploads/file1/20231018/652f331bc7aed.pdf
24. Thrasher, B., Wang, W., Michaelis, A., Melton, F., Lee, T., and Nemani, R. (2022). NASA Global Daily Downscaled Projections, CMIP6. Sci Data *9*, 262. https://doi.org/10.1038/s41597-022-01393-4.
25. NASA Earth Exchange Global Daily Downscaled Projections (NEX-GDDP-CMIP6) was accessed on 10-20-2024 from https://registry.opendata.aws/nex-gddp-cmip6.
26. Liu, L., He, G., Wu, M., Liu, G., Zhang, H., Chen, Y., Shen, J., and Li, S. (2023). Climate change impacts on planned supply–demand match in global wind and solar energy systems. Nat Energy *8*, 870–880. https://doi.org/10.1038/s41560-023-01304-w.
27. Cong, S., Nock, D., Qiu, Y.L., and Xing, B. (2022). Unveiling hidden energy poverty using the energy equity gap. Nat Commun *13*, 2456. https://doi.org/10.1038/s41467-022-30146-5.
28. Li, Y., Pizer, W.A., and Wu, L. (2019). Climate change and residential electricity consumption in the Yangtze River Delta, China. Proc. Natl. Acad. Sci. USA 116, 472–477. https://doi.org/10.1073/pnas.1804667115.
29. National Bureau of Statistics (NBS). China City Statistical Yearbook 2021. China Statistics Press.
30. National Development and Reform Commission. Typical power load curves in each provincial power system (NDRC). https://www.ndrc.gov.cn/xxgk/zcfb/tz/201912/P020191230336066090861.pdf (2019).
31. Zhuo, Z., Du, E., Zhang, N., Nielsen, C. P., Lu, X., Xiao, J., Wu, J., & Kang, C. (2022). Cost increase in the electricity supply to achieve carbon neutrality in China. *Nature Communications*, *13*(1), 1-13. https://doi.org/10.1038/s41467-022-30747-0
32. Fan, J.-L., Li, Z., Huang, X., Li, K., Zhang, X., Lu, X., Wu, J., Hubacek, K., and Shen, B. (2023). A net-zero emissions strategy for China's power sector using carbon-capture utilization and storage. Nat Commun *14*, 5972. https://doi.org/10.1038/s41467-023-41548-4.
33. China Atomic Energy Authority (CAEA). National nuclear power operation status from January to December 2021. https://www.caea.gov.cn/

34. Van Vliet, M. T., Yearsley, J. R., Ludwig, F., Vögele, S., Lettenmaier, D. P., and Kabat, P. (2012). Vulnerability of US and European electricity supply to climate change. Nat. Clim. Change 2(9), 676-681. https://www.nature.com/articles/nclimate1546.
35. Zhang, H., Da, Y., Zhang, X., and Fan, J.-L. (2021). The impacts of climate change on coal-fired power plants: evidence from China. Energy Environ. Sci. *14*, 4890–4902. https://doi.org/10.1039/D1EE01475G.
36. Henry, C.L., and Pratson, L.F. (2016). Effects of Environmental Temperature Change on the Efficiency of Coal- and Natural Gas-Fired Power Plants. Environ. Sci. Technol. *50*, 9764–9772. https://doi.org/10.1021/acs.est.6b01503.
37. Dumas, M., Kc, B., and Cunliff, C.I. (2019). Extreme Weather and Climate Vulnerabilities of the Electric Grid: A Summary of Environmental Sensitivity Quantification Methods. In, p. ORNL/TM-2019/1252, 1558514. https://doi.org/10.2172/1558514.
38. Liu, X., Tang, Q., Voisin, N., and Cui, H. (2016). Projected impacts of climate change on hydropower potential in China. Hydrology and Earth System Sciences *20*, 3343–3359. https://doi.org/10.5194/hess-20-3343-2016.
39. Förster, H., and Lilliestam, J. (2010). Modeling thermoelectric power generation in view of climate change. Reg Environ Change *10*, 327–338. https://doi.org/10.1007/s10113-009-0104-x.
40. Ji, J., Lu, J.-P., Chow, T.-T., He, W., and Pei, G. (2007). A sensitivity study of a hybrid photovoltaic/thermal water-heating system with natural circulation. Applied Energy *84*, 222–237. https://doi.org/10.1016/j.apenergy.2006.04.009.
41. Cavadini, G.B., and Cook, L.M. (2021). Green and cool roof choices integrated into rooftop solar energy modelling. Applied Energy *296*, 117082. https://doi.org/10.1016/j.apenergy.2021.117082.
42. Huang, J., Jones, B., Thatcher, M., and Landsberg, J. (2020). Temperature impacts on utility-scale solar photovoltaic and wind power generation output over Australia under RCP 8.5. Journal of Renewable and Sustainable Energy *12*, 046501. https://doi.org/10.1063/5.0012711.
43. Gao, Y., Ma, S., and Wang, T. (2019). The impact of climate change on wind power abundance and variability in China. Energy *189*, 116215. https://doi.org/10.1016/j.energy.2019.116215.
44. Gu, Y., Xu, J., Chen, D., Wang, Z., and Li, Q. (2016). Overall review of peak shaving for coal-fired power units in China. Renewable and Sustainable Energy Reviews *54*, 723–731. https://doi.org/10.1016/j.rser.2015.10.052.
45. Ritchie, H. (2024). China is building more coal plants but might burn less coal. https://www.sustainabilitybynumbers.com/p/china-coal-plants.
46. Chen, S., Chen, Y., Lei, Z., and Tan-Soo, J.-S. (2021). Chasing Clean Air: Pollution-Induced Travels in China. Journal of the Association of Environmental and Resource Economists *8*, 59–89.
47. Yalew, S.G., van Vliet, M.T.H., Gernaat, D.E.H.J., Ludwig, F., Miara, A., Park, C., Byers, E., De Cian, E., Piontek, F., Iyer, G., et al. (2020). Impacts of climate change on energy systems in global and regional scenarios. Nat Energy *5*, 794–802. https://doi.org/10.1038/s41560-020-0664-z.
48. Zhang, S., Guo, Q., Smyth, R., and Yao, Y. (2022). Extreme temperatures and residential electricity consumption: Evidence from Chinese households. Energy Economics *107*, 105890. https://doi.org/10.1016/j.eneco.2022.105890.

49. Gernaat, D.E.H.J., de Boer, H.S., Daioglou, V., Yalew, S.G., Müller, C., and van Vuuren, D.P. (2021). Climate change impacts on renewable energy supply. Nat. Clim. Chang. *11*, 119–125. https://doi.org/10.1038/s41558-020-00949-9.
50. Abdin, A.F., Fang, Y.-P., and Zio, E. (2019). A modeling and optimization framework for power systems design with operational flexibility and resilience against extreme heat waves and drought events. Renewable and Sustainable Energy Reviews *112*, 706–719. https://doi.org/10.1016/j.rser.2019.06.006.
51. Schmietendorf, K., Peinke, J., and Kamps, O. (2017). The impact of turbulent renewable energy production on power grid stability and quality. Eur. Phys. J. B *90*, 222. https://doi.org/10.1140/epjb/e2017-80352-8.
